# Ankle-brachial index and ocular diseases in a Russian population

**DOI:** 10.1038/s41433-021-01846-x

**Published:** 2021-11-29

**Authors:** Mukharram M. Bikbov, Timur R. Gilmanshin, Rinat M. Zainullin, Ellina M. Rakhimova, Iuliia A. Rusakova, Albina A. Fakhretdinova, Azaliia M. Tuliakova, Gyulli M. Kazakbaeva, Songhomitra Panda-Jonas, Kamilia R. Safiullina, Nataliia I. Bolshakova, Ainur V. Gizzatov, Ildar P. Ponomarev, Nikolai A. Nikitin, Nail E. Baimukhametov, Jost B. Jonas

**Affiliations:** 1 Ufa Eye Research Institute, Ufa, Bashkortostan Russia; 2 Privatpraxis Prof Jonas und Dr Panda-Jonas, Heidelberg, Germany; 3 Department of Ophthalmology, Medical Faculty Mannheim of the Ruprecht-Karls-University of Heidelberg, Mannheim, Germany; 4 Institute of Molecular and Clinical Ophthalmology Basel, Basel, Switzerland

**Keywords:** Retinal diseases, Lens diseases

## Abstract

**Background:**

To assess potential associations between the ankle-brachial blood pressure index (ABI) and ocular disorders.

**Methods:**

In the population-based cross-sectional Russian Ural Eye and Medical Study including 5,899 (80.5%) out of 7328 eligible participants aged 40+ years, the participants underwent a series of ocular and medical examinations including measurement of ABI.

**Results:**

Blood pressure measurements of both arms and ankles were available for 3187 (54.0%) individuals. The mean ABI was 1.26 ± 0.19 (median:1.20; range: 0.61, 2.20). In multivariate analysis, a higher ABI was associated with younger age (*P* < 0.001; non-standardized regression coefficient B: −0.001; 95% confidence interval (CI): −0.002, −0.001), female sex (*P* < 0.001; B: 0.03; 95% CI: 0.02, 0.04), lower body mass index (*P* < 0.001; B: −0.004; 95% CI: −0.006, −0.003), lower waist-to-hip ratio (*P* = 0.01; B: −0.10; 95% CI: −0.17, −0.02), lower glucose serum concentration (*P* = 0.008; B: −0.005; 95% CI: −0.009, −0.001), lower prevalence of arterial hypertension (*P* < 0.001; B: −0.14; 95% CI: −0.16, −0.12), higher mean systolic blood pressure (*P* < 0.001; B: 0.003; 95% CI: 0.002, 0.003), and higher prevalence of any alcohol consumption (*P* < 0.001; B: 0.03; 95% CI: 0.02, 0.04). In that multivariate model, prevalence of glaucoma (*P* = 0.67) as a whole, open-angle glaucoma (*P* = 0.86) and angle-closure glaucoma (*P* = 0.54), stage of glaucomatous optic neuropathy (*P* = 0.57), prevalence of age-related macular degeneration (*P* = 0.88), prevalence and stage of diabetic retinopathy (*P* = 0.30, and *P* = 0.29, respectively), nuclear cataract (*P* = 0.32, and *P* = 0.41, resp.), cortical cataract (*P* = 0.33, and *P* = 0.92, resp.), subcapsular cataract (*P* = 0.74 and *P* = 0.60, resp.), and pseudoexfoliation (*P* = 0.44 and *P* = 0.47, resp.), intraocular pressure (*P* = 0.52), axial length (*P* = 0.20), and peripapillary retinal nerve fibre layer thickness (*P* = 0.55) were not significantly associated with the ABI.

**Conclusions:**

In this ethnically mixed population from Russia, none of the major ocular diseases was associated with ABI suggesting that subclinical atherosclerosis is not markedly associated with the aetiology of these ocular disorders.

## Introduction

Arteriosclerosis is a sign of arterial aging and, due to a thickening, hardening and loss of elasticity of the arterial walls, gradually reduces the blood flow. It is a major risk factor for arterial hypertension and cardiovascular and cerebrovascular disease. The hardening of the arterial walls in arteriosclerosis leads to a loss of the buffering effect of the Windkessel function on systolic blood pressure (BP). The resulting premature return of the counter-pulsation to the aorta increases the systolic BP, decreases the diastolic BP, and increases the pulse pressure (PP). It results in damage to low-resistance organs including the heart, brain, kidney, and liver [1]. The risk of cardiovascular and cerebrovascular events, cognitive dysfunction, renal dysfunction, fatty liver, cardiovascular disease, and all-cause death increases with arteriosclerosis [1–5].

Despite the importance of atherosclerosis for cardiovascular and cerebrovascular diseases and although the retinal and choroidal blood perfusion of the eye is part of the cerebrovascular system, only a few studies have addressed the question whether atherosclerosis affects the prevalence of ocular diseases [6–16]. These studies had limitations such as including a relatively small number of participants, a hospital-based recruitment of the study participants, and in particular, not addressing most of the systemic parameters influencing the measurements of atherosclerosis so that confounding effects might have occurred. We, therefore, conducted this study to investigate the association between arteriosclerosis and ocular parameters in a relatively large study population recruited on a population-basis and examining a relatively large number of systemic factors which may influence atherosclerosis. Arteriosclerosis was estimated by determination of the ankle-brachial blood pressure index (ABI) defined as the ratio of the ankle to the brachial systolic BP. It is an indicator of atherosclerotic vascular disease in the lower extremities and a simple, non-invasive measure of subclinical atherosclerosis in general [17–21].

## Patients and methods

The UEMS is a population-based investigation that was performed in the Russian republic of Bashkortostan at the southwestern end of the Ural Mountains in the study period from 2015 to 2017 [22, 23]. Study regions were Ufa as capital of Bashkortostan in a distance of about 1400 km East of Moscow and a rural region in the Karmaskalinsky District at a distance of 65 km from Ufa. The republic of Bashkortostan located between the Volga River and the Ural Mountains, is with a population of four million people the most populous republic in Russia. Inclusion criteria for the study were living in the study regions and an age of 40 years or older. The Ethics Committee of the Academic Council of the Ufa Eye Research Institute approved the study design and confirmed that the study adhered to the Declaration of Helsinki, and all participants gave informed written consent.

Using a bus, the study participants were brought from their homes to the Ufa Eye Institute where a team of about 20 trained and surveyed technicians and ophthalmologists performed all examinations. The series of examinations started with a detailed interview performed by trained social workers. It consisted of more than 250 standardized questions on the socioeconomic background, smoking habits and alcohol consumption, physical activity, diet, depression and anxiety, and known diagnosis and therapy of major diseases. The examinations further included anthropometry, BP measurement, handgrip dynamometry, spirometry, and biochemical analysis of blood samples taken under fasting conditions. The BP at the arm was determined with the individual sitting for at least five minutes. The individuals had not smoked or taken any coffee, tea or alcohol for at least three hours, nor had they undertaken any physical exercise for half an hour before the BP measurements were taken. We measured the BP three times, and we took the mean value for further statistical analysis. We used an automatic tonometer (OMRON M2, Omron Co. Kyoto, Japan) the cuff size of which was adapted to the upper arm circumference. The BP at the ankle was measured after the participants had rested in the lying position for at least five minutes. Again, their measurements were obtained, the mean value of which was taken for further analysis. We calculated the ABI as the ratio of the highest BP values of both ankles divided by the highest BP values of both arms. Additionally, we calculated the right ABI as the ratio of the BP at the right ankle divided by the highest BP values of both arms, and the left ABI as the ratio of the BP at the left ankle divided by the highest BP values of both arms. The estimated glomerular filtration rate (eGFR) was calculated using the Chronic-Kidney-Disease-Epidemiology-Collaboration (CKD-EPI) equation [24]. We applied the Guidelines for Accurate and Transparent Health Estimates Reporting (GATHER statement guidelines) for collecting the data [25]. According to the new criteria published by the American Heart Association, we differentiated between normal BP, elevated BP, stage 1 and stage 2 of arterial hypertension, and a hypertensive crisis [26]. Diagnostic criteria for diabetes mellitus were a fasting serum glucose concentration of ≥7.0 mmol/L or a self-reported history of physician-based diagnosis or therapy of diabetes mellitus. We described the study design in detail recently.

The ophthalmologic examinations included measurement of visual acuity included automated and subjective refractometry (Auto-2Ref/Keratometer HRK-7000A HUVITZ Co, Ltd., Gyeonggi-do, Korea), perimetry (PTS 1000 Perimeter, Optopol Technology Co., Zawercie, Poland), Scheimflug imaging (Pentacam HR, Typ70900, OCULUS, Optikgeräte GmbH Co., Wetzlar, Germany) of the anterior segment, slit lamp-based biomicroscopy of the anterior and posterior ocular segment, non-contact tonometry (Tonometer Kowa KT-800, Kowa Company Ltd., Hamamatsu City, Japan), re-assessment of the anterior segment and lens for the presence of pseudoexfoliation after medical mydriasis, photography of the cornea and lens (Topcon slit lamp and camera, Topcon Corp. Tokyo, Japan), optical coherence tomography (OCT) ((RS-3000, NIDEK co., Ltd., Aichi Japan) of the peripapillary retinal nerve fibre layer, optic nerve head, and macula, and assessment of the degree of fundus tessellation using the fundus photographs. Nuclear lens opacities were differentiated into six grades using the classifying scheme for cataracts of the Age-Related Eye Disease Study [27]. We defined the presence of nuclear cataract as a nuclear cataract grade of three or higher. Cortical lens opacities and posterior subcapsular opacities were graded using photographs taken by retro-illumination (Topcon slit lamp and camera, Topcon Corp. Tokyo, Japan). Using a grid, we measured the percentage area of opacity. Age-related macular degeneration (AMD) was defined as suggested by the recent Beckman Initiative for Macular Research Classification Committee [28]. Glaucoma was defined by morphological criteria as described by Foster and colleagues [29].

Inclusion criterion for the present study was the availability of BP measurements at both arms and both ankles as the basis for the calculation of the ABI. The data were statistically analysed using a statistical software package (SPSS for Windows, version 25.0, IBM-SPSS, Chicago, IL, USA). We assessed the mean values of the parameters (expressed as mean and standard deviation or as mean and 95% confidence intervals (CI)) and examined associations between the ABI and other systemic parameters and ocular parameters, first in a univariate analysis, followed by multivariable analysis. The latter included the ABI as the dependent variable and as independent parameters all those variables that were associated (*P* ≤ 0.10) with the ABI in the univariate analyses. Out of the list of independent variables, we then dropped parameters due to collinearity with other independent variables (with a cut-off value of the variance inflation factor (VIF) of 2.0 or higher) or if they were no longer significantly associated with the ABI. We calculated odds ratios (OR) and their 95% CI. All *P* values were two-sided and considered statistically significant when the values were less than 0.05.

## Results

The Ural Eye and Medical Study consisted of 5899 individuals out of whom the ABI had been measured for 3187 (54.0%) individuals (1765 (55.4%) men). From an ethnic point of view, there were 597 (18.7%) individuals of Russian ethnicity, 533 (16.7%) Bashkirs, 1753 (55.0%) Tartars, 222 (7.0%) Chuvash, 16 (0.5%) Mari, and 66 (2.1%) individuals of other or undeclared ethnic background. The study population with a mean age of 57.3 ± 9.7 years (median: 56 years; range: 40–90 years) showed a similar distribution of age and gender as the population of Russia beyond an age of 40+ years and examined in the census of 2010. Both showed a preponderance of women and two constrictions in the age distribution due to the consequences of World War II [30, 31]. The study population as compared to the total population of Russia showed a higher proportion of Tartars (3.7% in whole Russia) and Bashkirs (1.1% in whole Russia), and correspondingly, a lower proportion of Russians (77.7% in whole Russia). Age was significantly younger (57.3 ± 9.7 years versus 60.9 ± 11.5 years; *P* < 0.001), axial length longer (24.3 ± 1.1 mm versus 23.2 ± 1.1 mm; *P* < 0.001) and the percentage of men higher (men/women: 1765/1422 versus 815/1897; *P* < 0.001). in the group of individuals with ABI measurements than in group without ABI data.

**Fig. 1:**
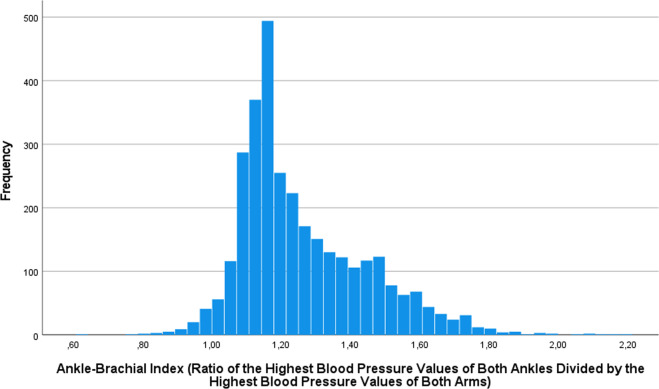
Distribution of the Ankle-Brachial Index. Histogram showing the distribution of the ankle-brachial index in the population of the Ural Eye and Medical Study.

The mean systolic/diastolic BP was 133.5 ± 19.6 mmHg/82.6 ± 10.3 mmHg for the right arm and 133.3 ± 20.0 mmHg/82.1 ± 11.0 mmHg for the left arm (Table 1). It was 168.0 ± 24.5 mmHg/101.8 ± 12.7 mmHg for the right ankle, and 166.6 ± 24.6 mmHg/100.0 ± 12.9 mmHg for the left ankle. The mean right ABI was 1.25 ± 0.19 (median: 1.19; range: 0.60, 2.20), the mean left ABI was 1.24 ± 0.19 (median: 1.18; range: 0.61, 2.17), and the mean ABI calculated using the highest values of both sides was 1.26 ± 0.19 (median: 1.20; range: 0.61, 2.20) (Fig. 1).

**Table 1:** Mean values and associations (univariate analysis) between the ankle brachial index (defined as the ratio of the highest values of ankle systolic blood pressure of both sides divided by the highest value of arm systolic blood pressure of both sides) and systemic parameters in the Ural Eye and Medical Study.

Parameter	Interval	*n*	Mean	Non-standardized Regression coefficient B	95% Confidence Interval of B	*P* Value
Age	1-year intervals	3,187	57.3 ± 9.7	−0.001	−0.002, 0.000	0.006
Gender	Men/Women	3,187	1765/1422	0.04	0.03, 0.05	<0.001
Region of habitation	Urban/Rural	3,187	973/2214	−0.01	−0.03, 0.003	0.11
Ethnicity	Non-Russian ethnicity/Russian	3,187	2590/597	−0.01	−0.03, 0.01	0.18
Body height	1 cm	3,187	166.4 ± 8.8	−0.002	−0.002, −0.001	<0.001
Body weight	kg	3,187	76.9 ± 14.6	−0.002	−0.002, −0.001	<0.001
Body mass index	kg/m^2^	3,187	27.7 ± 4.8	−0.004	−0.005, −0.003	<0.001
Waist circumference	cm	3,187	93.9 ± 13.4	−0.002	−0.002, −0.001	<0.001
Hip circumference	cm	3,187	102.6 ± 12.8	−0.001	−0.001, 0.000	0.002
Waist/hip circumference ratio	Ratio	3,187	0.92 ± 0.09	−0.25	−0.32, −0.18	<0.001
Socioeconomic Score	Score	3,185	5.94 ± 1.24	0.009	0.004, 0.014	0.001
Level of education	Illiteracy/Passing 5th grade/8th grade/10th grade/11th grade/Graduates/Specialized secondary education/Post graduates	3,187	5.8 ± 1.2	0.005	0.000, 0.010	0.05
Physical activity Score	Score	3,156	9.1 ± 8.4	0.001	0.000, 0.002	0.02
Smoking, currently	Yes/No	3,186	459/2727	−0.006	−0.02, 0.01	0.54
Smoking, package years	Number	3,146	4.6 ± 13.1	0.000	−0.001, 0.000	0.56
Alcohol consumption, any	Yes/No	3,187	923/2264	0.02	0.005, 0.03	0.007
In a week how many days do you eat fruits?	Number of days	3,167	5.6 ± 1.8	0.002	−0.002, 0.006	0.27
In a week how many days do you eat vegetables?	Number of days	3,183	6.4 ± 1.2	0.005	0.000, 0.010	0.06
History of cardiovascular disorders including stroke	Yes/No	3,187	792/2,395	−0.003	−0.02, 0.01	0.67
History of angina pectoris	Yes/No	3,187	275/2,912	0.03	0.002, 0.05	0.03
History of asthma	Yes/No	3,187	66/3,121	−0.002	−0.05, 0.04	0.94
History of arthritis	Yes/No	3,187	802/2,385	0.01	−0.003, 0.03	0.12
History of previous bone fractures	Yes/No	3,187	952/2,235	0.005	−0.01, 0.02	0.46
History of low back pain	Yes/No	3,187	1,627/1,560	0.02	0.004, 0.03	0.009
History of thoracic spine pain	Yes/No	3,187	695/2,492	0.02	0.004, 0.04	0.02
History of neck pain	Yes/No	3,187	892/2,295	0.02	0.002, 0.30	0.03
History of headache	Yes/No	3,187	1,466/1,721	0.02	0.004, 0.03	0.01
History of cancer	Yes/No	3,187	77/3,110	0.000	−0.04, 0.04	0.99
History of dementia	Yes/No	3,187	13/3,174	−0.04	−0.14, 0.06	0.47
History of diarrhea	Yes/No	3,187	10/3,177	0.15	0.03, 0.27	0.01
History of iron-deficiency anemia	Yes/No	3,187	141/3,046	0.08	0.05, 0.11	<0.001
History of low blood pressure and hospital admittance	Yes/No	3,176	98/3,078	−0.01	−0.05, 0.02	0.45
History of osteoarthritis	Yes/No	3,187	593/2594	0.03	0.008, 0.04	0.003
History of skin disease	Yes/No	3,187	147/3,040	0.02	−0.01, 0.05	0.20
History of thyroid disease	Yes/No	3,187	228/2,959	0.03	0.01, 0.06	0.01
History of falls	Yes/No	3,187	513/2,674	0.01	−0.01, 0.02	0.50
History of unconsciousness	Yes/No	3,187	206/2,981	0.003	−0.02, 0.03	0.83
Age of the last menstrual bleeding	Years	1,121	48.0 ± 5.2	−0.001	−0.003, 0.001	0.39
Age of last regular menstrual bleeding	Years	1,123	47.9 ± 5.2	−0.001	−0.003, 0.001	0.28
History of menopause	Yes/No	1,409	1125/284	−0.04	−0.07, −0.01	0.003
Serum concentration of:						
Alanine aminotransferase	IU/L	3,165	20.4 ± 11.0	−0.001	−0.002, −0.001	<0.001
Aspartate aminotransferase	IU/L	3,169	20.4 ± 9.6	−0.002	−0.002, −0.001	<0.001
Aspartate aminotransferase-to- Alanine aminotransferase ratio	Ratio	3,164	1.07 ± 0.51	−0.004	−0.02, 0.01	0.55
Bilirubin, total	µmol/L	3,170	16.2 ± 11.6	0.000	0.000, 0.001	0.69
High-density lipoproteins	mmol/L	3,170	2.23 ± 0.78	−0.002	−0.01, 0.01	0.68
Low-density lipoproteins	mmol/L	3,170	2.13 ± 1.08	−0.001	−0.01, 0.01	0.86
Cholesterol	mmol/L	3,170	5.70 ± 1.37	−0.001	−0.01, 0.003	0.59
Triglycerides	mmol/L	3,169	1,37 ± 0.67	−0.001	−0.01, 0.01	0.78
Rheumatoid factor	IU/mL	3,170	0.1 ± 0.3	−0.01	−0.03, 0.01	0.55
Erythrocyte sedimentation rate	Mm/min	3,170	13.4 ± 11.3	0.001	0.00, 0.002	0.001
Glucose	mmol/L	3,170	4.99 ± 1.71	−0.01	−0.01, −0.003	<0.001
Urea	mmol/L	3,170	4.72 ± 1.36	−0.01	−0.01, −0.002	0.003
Creatinine	µmol/L	3,161	93.0 ± 21.7	0.000	−0.001, 0.000	0.002
Hemoglobin	g/L	3,170	144.7 ± 14.6	−0.001	−0.001, 0.000	<0.001
Erythrocyte count	10^6^ cells/µL	3,170	4.55 ± 0.38	−0.04	−0.05, −0.02	<0.001
Leukocyte count	10^9^ cells/L	3,170	5.14 ± 1.44	0.000	−0.005, 0.005	1.00
Prevalence of diabetes mellitus	Yes/No			−0.02	−0.04, 0.002	0.08
Estimated glomerular filtration rate	30 mL/min/1.73 m²	3,161	71.8 ± 16.8	0.001	0.000, 0.001	0.006
Stage of chronic kidney disease	0–5	3,161	21.4 ± 6.8	−0.001	−0.002, 0.000	0.04
Anemia	Yes/No	3,170	220/2950	0.03	0.000, 0.05	0.049
Blood pressure, systolic, right arm	mm Hg	3,187	133.5 ± 20.0	−0.004	−0.004, −0.004	<0.001
Blood pressure, systolic, left arm	mm Hg	3,187	133.3 ± 20.0	−0.004	−0.004, −0.004	<0.001
Blood pressure, systolic, right ankle	mm Hg	3,187	168.0 ± 24.5	0.004	0.004, 0.004	<0.001
Blood pressure, systolic, left ankle	mm Hg	3,187	166.6 ± 24.6	0.004	0.004, 0.004	<0.001
Blood pressure, diastolic, right arm	mm Hg	3,187	82.6 ± 10.3	−0.005	−0.006, −0.005	<0.001
Blood pressure, diastolic, left arm	mm Hg	3,187	82.1 ± 11.0	−0.005	−0.005, −0.004	<0.001
Blood pressure, diastolic, right ankle	mm Hg	3,187	101.8 ± 12.7	0.005	0.005, 0.006	<0.001
Blood pressure, diastolic, left ankle	mm Hg	3,187	100.9 ± 12.9	0.006	0.005, 0.006	<0.001
Mean systolic blood pressure (mean of both arms and both legs)	mm Hg	3,187	150.3 ± 19.2	0.001	0.001, 0.001	<0.001
Mean diastolic blood pressure (mean of both arms and both legs)	mm Hg	3,187	91.8 ± 9.2	0.002	0.001, 0.003	<0.001
Arterial hypertension	Yes/No	3,187	2788/399	−0.14	−0.16, −0.12	<0.001
Arterial hypertension, stage	0–4	3,187	2.2 ± 1.0	−0.06	−0.07, −0.06	<0.001
Prevalence of chronic obstructive pulmonary disease	Yes/No	3,183	161/3022	0.02	−0.01, 0.05	0.14
Hearing loss	Hearing loss score (0–44)	3,187	5.50 ± 11.9	0.000	0.00, 0.001	0.32
Depression Score	Depression score unit (range: −4 to +15)	3,186	0.68 ± 3.69	0.001	0.000, 0.003	0.09
State-trait anxiety inventory	State-trait anxiety inventory score (range: −7 to 13)	3,185	−1.09 ± 3.39	0.000	−0.002, 0.002	0.99
Manual dynamometry, right hand	dekaNewton	3,180	33.1 ± 12.0	−0.001	−0.002, 0.000	<0.001
Manual dynamometry, right hand	dekaNewton	3,179	29.6 ± 11.6	−0.001	−0.002, −0.001	<0.001

In univariate analysis, a higher ABI was associated with the systemic parameters of female gender (*P* < 0.001), higher mean systolic BP (mean of both arms and both ankles) (*P* < 0.001), higher mean diastolic BP (mean of both arms and both ankles) (*P* < 0.001), a higher socioeconomic score (*P* = 0.001) and higher level of education (*P* = 0.05), higher physical activity score (*P* = 0.02), higher prevalence of any alcohol consumption (*P* = 0.007), higher number of days with vegetable intake, (*P* = 0.06), higher frequency of a positive history of angina pectoris (*P* = 0.03), back pain (*P* = 0.009), thoracic spine pain (*P* = 0.02), neck pain (*P* = 0.03) and headache (*P* = 0.01), diarrhoea (*P* = 0.01), iron-deficiency anaemia (*P* < 0.001), osteoarthritis (*P* = 0.003), and thyroid disorder (*P* = 0.01), higher erythrocyte sedimentation rate (*P* < 0.001), higher eGFR (*P* = 0.006), higher prevalence of anaemia (*P* = 0.049), and a higher depression score (*P* = 0.09) (Table 1). A higher ABI was correlated with younger age (*P* = 0.006), lower body height (*P* < 0.001), body weight (*P* < 0.001) and body mass index (*P* < 0.001), shorter waist (*P* < 0.001) and hip circumference (*P* < 0.001) and lower waist-to-hip circumference ratio (*P* = 0.002), a lower serum concentrations of alanine aminotransferase (*P* < 0.001), aspartate aminotransferase (*P* < 0.001), glucose (*P* < 0.001), urea (*P* = 0.003), creatinine (*P* = 0.002), and haemoglobin (*P* < 0.001), lower erythrocyte count (*P* < 0.001), lower stage of chronic kidney disease (*P* = 0.04), lower prevalence of diabetes mellitus (*P* = 0.08) and arterial hypertension (*P* < 0.001), and lower dynamometric hand force (*P* < 0.001) (Table 1). A higher ABI was associated with the ocular parameters of larger lens thickness (*P* = 0.047), and with a lower degree of fundus tessellation in the macular region (*P* = 0.02) and peripapillary region (*P* = 0.048), and lower foveal retinal thickness (*P* = 0.001) (Table 2).

**Table 2:** Mean values and associations (univariate analysis) between the ankle brachial index (defined as the ratio of the highest values of ankle systolic blood pressure of both sides divided by the highest value of arm systolic blood pressure of both sides) and ocular parameters in the Ural Eye and Medical Study.

Parameter	Interval	*n*	Mean	Non-standardized regression coefficient B	95% Confidence interval of B	*P*-Value
Refractive error, spherical equivalent	Diopters	3,026	0.23 ± 2.16	0.000	−0.003, 0.004	0.75
Refractive error, cylindrical value	Diopters	3,056	−0.70 ± 0.76	0.002	−0.007, 0.011	0.64
Axial length	mm	3,148	23.4 ± 1.0	−0.001	−0.007, 0.006	0.87
Corneal refractive power	Diopters	3,149	43.7 ± 1.7	0.004	0.000, 0.008	0.06
Central corneal thickness	µm	3,150	541.9 ± 33.7	0.000	0.000, 0.000	0.92
Corneal volume	mm^3^	3,151	59.2 ± 3.9	0.001	−0.001, 0.002	0.44
Anterior chamber depth	mm	3,146	3.20 ± 0.46	0.000	−0.015, 0.014	0.96
Anterior chamber volume	µL	3,151	131.8 ± 34.4	0.000	0.000, 0.000	0.36
Anterior chamber angle	Degree	3,146	31.9 ± 6.6	−0.001	−0.002, 0.000	0.19
Lens thickness	mm	3,048	4.57 ± 0.41	0.016	0.000, 0.033	0.047
Nuclear cataract degree	Grade	3,044	2.05 ± 1.01	−0.004	−0.010, 0.003	0.28
Nuclear cataract, presence	Yes/No	3,044	963/2081	−0.12	−0.026, 0.002	0.09
Cortical cataract, degree	Percentage	3,043	2.24 ± 8.8	0.000	−0.001, 0.000	0.45
Cortical cataract, presence	Yes/No	3,043	328/2715	0.002	−0.019, 0.024	0.83
Subcapsular cataract, degree	Percentage	3,045	0.14 ± 2.02	0.001	−0.003, 0.004	0.70
Age-related macular degeneration	Yes/No	2,566	267/2,299	0.004	−0.01, 0.01	0.41
Age-related macular degeneration	No/early/Intermediate/Late	2,566	2,299/195/59/13	0.001	−0.01, 0.01	0.72
Subcapsular cataract, presence	Yes/No	3,045	17/3028	0.019	−0.070, 0.107	0.68
Fundus tessellation, macula region	Grade	2,558	0.60 ± .86	−0.01	−0.19, 0.002	0.02
Fundus tessellation, peripapillary region	Grade	2,981	1.10 ± 0.97	−0.01	−0.014, 0.000	0.048
Intraocular pressure,	mmHg	3,143	13.5 ± 4.0	−0.001-	0.002, 0.001	0.33
Retinal thickness (total), fovea	µm	3,096	230 ± 44	0.000	0.000, 0.000	0.08
Retinal thickness (total), 300 µm temporal to the fovea	µm	3,096	258 ± 36	0.000	−0.001, 0.000	0.001
Retinal thickness (total), 300 µm nasal to the fovea	µm	3,095	261 ± 41	0.000	0.000, 0.000	0.14
Retinal nerve fiber layer thickness	µm	3,089	115.2 ± 18.5	0.000	0.000, 0.001	0.34
Glaucoma	Yes/No	3,049	98/2591	−0.002	−0.039, 0.036	0.93
Glaucoma stage	0–5	3,049	0.07 ± 0.43	0.000	−0.015, 0.016	0.98
Open-angle glaucoma	Yes/No	3,049	65/2,984	−0.001	−0.047, 0.045	0.97
Angle-closure glaucoma	Yes/No	3,049	33/3,016	0.003	−0.061, 0.067	0.92
Diabetic retinopathy	Yes/No	2,557	41/2,516	−0.049	−0.107, 0.009	0.095
Diabetic retinopathy, ETDRS grading	Scale	2,559	0.03 ± 0.22	−0.027	−0.060, 0.006	0.10

**Fig. 2:**
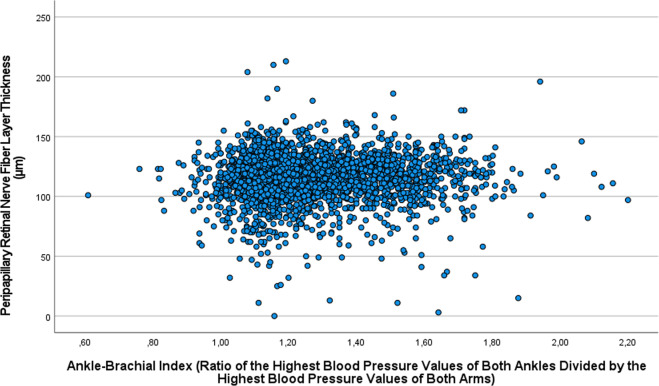
Distribution of the Ankle-Brachial Index in Association with the Peripapillary Retinal Nerve Fiber Layer Thickness. Scattergram showing the distribution of the ankle-brachial index in dependence of the peripapillary retinal nerve fibre layer thickness in the population of the Ural Eye and Medical Study.

In the multivariable analysis with the ABI as the dependent variable and with all those parameters, which were significantly associated with ABI in the univariate analysis, as independent variables, we first dropped in a step-by-step manner parameters due to collinearity and then parameters which were no longer significantly associated with ABI. In the final model, a higher ABI was associated (correlation coefficient r: 0.38) with younger age (*P* < 0.001), female sex (*P* < 0.001), lower body mass index (*P* < 0.001), lower waist-to-hip ratio (*P* = 0.01), lower glucose serum concentration (*P* = 0.008), lower prevalence of arterial hypertension (*P* < 0.001), higher mean systolic BP (*P* < 0.001), higher prevalence of a history diarrhoea (*P* = 0.02), iron-deficiency anaemia (*P* = 0.02) and osteoarthritis (*P* = 0.01), and higher prevalence of any alcohol consumption (*P* < 0.001) (Table 3, Supplementary Table 1). When the ocular parameters of prevalence of glaucoma, prevalence of open-angle glaucoma, prevalence of angle-closure glaucoma, stage of glaucomatous optic neuropathy, prevalence of age-related macular degeneration, prevalence of diabetic retinopathy, stage of diabetic retinopathy, prevalence of nuclear cataract, degree of nuclear cataract, prevalence of cortical cataract, degree of cortical cataract, prevalence of subcapsular cataract, degree of subcapsular cataract, intraocular pressure, axial length, peripapillary retinal nerve fibre layer thickness (Fig. 2), and prevalence and stage of pseudoexfoliation, were added as single parameters to the model, none of them were significantly associated with the ABI. If the serum glucose concentration was dropped from the model, a lower ABI was associated with a higher prevalence of diabetic retinopathy (B: −0.06; 95% CI: −0.12, −0.004; *P* = 0.035).

**Table 3:** Associations (multivariate analysis) between the ankle brachial index (ABI) (defined as the ratio of the highest values of ankle systolic blood pressure of both sides divided by the highest value of arm systolic blood pressure of both sides) and ocular parameters in the Ural Eye and Medical Study.

Parameter	Interval	Non-standardized regression coefficient B	95% confidence interval of B	*P* Value	Variance inflation factor (VIF)
Age	Years	−0.001	−0.002, −0.001	<0.001	1.17
Sex	Men - Women	0.03	0.02, 0.04	<0.001	1.29
Body mass index	kg/m^2^	−0.004	−0.006, −0.003	<0.001	1.26
Waist-hip circumference ratio	Ratio	−0.10	−0.17, −0.02	0.01	1.26
Glucose serum concentration	mmol/L	−0.005	−0.009, −0.001	0.008	1.05
Arterial hypertension, prevalence	Prevalence	−0.14	−0.16, −0.12	<0.001	1.27
Mean systolic blood pressure (mean of measurements at both arms and both ankles)	mmHg	0.003	0.002, 0.003	<0.001	1.37
History of diarrhea	Prevalence	0.13	0.02, 0.24	0.02	1.03
History iron-deficiency anemia	Prevalence	0.05	0.02, 0.08	0.02	1.05
History of osteoarthritis	Prevalence	0.02	0.01, 0.04	0.01	1.05
Alcohol consumption, any	Prevalence	0.03	0.02, 0.04	<0.001	1.07

When the ocular parameters of prevalence of glaucoma (*P* = 0.67), prevalence of open-angle glaucoma (*P* = 0.86), prevalence of angle-closure glaucoma (*P* = 0.54), stage of glaucomatous optic neuropathy (*P* = 0.57), prevalence of age-related macular degeneration (*P* = 0.88), prevalence of diabetic retinopathy (*P* = 0.30), stage of diabetic retinopathy (*P* = 0.29), prevalence of nuclear cataract (*P* = 0.32), degree of nuclear cataract (*P* = 0.41), prevalence of cortical cataract (*P* = 0.33), degree of cortical cataract (*P* = 0.92), prevalence of subcapsular cataract (*P* = 0.74), degree of subcapsular cataract (*P* = 0.60), intraocular pressure (*P* = 0.52), axial length (*P* = 0.20), peripapillary retinal nerve fiber layer thickness (*P* = 0.55), and prevalence (*P* = 0.44) and stage (*P* = 0.47) of pseudoexfoliation, were added as single parameters to the model, none of them were significantly associated with the ABI.

Comparable results were obtained if the right ABI or the left ABI were taken as the dependent variable. In a similar manner, the prevalence of an abnormal ABI defined by cut-off values of ≤0.99 and ≥1.3 was associated in a logistic binary regression analysis with the same independent parameters as was the ABI in the linear regression analysis.

## Discussion

In our Russian study population with an age of 40+ years, the mean ABI as ratio of the highest systolic values of both arms divided by the highest systolic values of both ankles was 1.26 ± 0.19 (median:1.20; range:0.61, 2.20). The ABI was not significantly associated in multivariable analysis with any major ocular disorder or parameter tested, including prevalence and stage of glaucoma as a whole and differentiated into open-angle glaucoma and angle-closure glaucoma, prevalence and stage of age-related macular degeneration, pseudoexfoliation, nuclear cataract, cortical cataract and subcapsular cataract, intraocular pressure, axial length, and peripapillary retinal nerve fibre layer thickness. A potential exception may have been diabetic retinopathy, the prevalence of which was associated with a lower ABI, if the serum glucose concentration was dropped from the multivariable model. Since however diabetic retinopathy might have just been a proxy for serum glucose concentration, it has remained elusive whether that ABI was indeed correlated with the diabetic retinopathy prevalence. The results suggested that subclinical atherosclerosis is not markedly associated with the prevalence of any of the major ocular diseases and ophthalmological parameters tested.

The results of our study agree with observations made in previous non-ophthalmological investigations in which a lower ABI correlated with older age, higher prevalence and stage of diabetes and arterial hypertension, higher serum cholesterol concentration, and other risk factors for cardiovascular and cerebrovascular disease [19–21]. The findings made in our study may also conform to the results of previous studies on an association between the ABI and the prevalence of diabetic retinopathy. In the Multi-Ethnic Study of Atherosclerosis on 927 individuals with diabetes and without clinical cardiovascular disease, the prevalence of vision-threatening diabetic retinopathy was associated with a low ABI and high coronary artery calcium score. It was associated with a high ABI after adjusting for serum haemoglobin A1c concentration and duration of diabetes [8]. In a hospital-based study conducted by Chen and colleagues on patients with a diabetes duration of longer than 10 years, an elevated serum HbA1c level (≥7.5%) and with an eGFR of ≥60 mL/min/1.73 m^2^, a higher prevalence of proliferative diabetic retinopathy was associated with a higher prevalence of low ABI (≤0.99) or high ABI (≥1.3) [13]. As in our study, Chang and associates reported that IOP was not associated with measures of subclinical atherosclerosis including brachial-ankle pulse wave velocity, ABI and vertebral artery flow [7].

The observations made in our study are partially contradictory to those reported by some previous investigations. Jeganathan and colleagues found in the Singapore Malay Eye Study that a lower ABI as an indicator of peripheral artery disease was related to glaucoma, supporting an association between large-vessel atherosclerotic disease and glaucoma [6]. Out of the 922 study participants of the Singapore Malay Eye Study, 79 (8.6%) had peripheral arterial disease (defined as an ABI < 0.9) and 42 (4.6%) had glaucoma, with individuals with peripheral arterial disease more likely having glaucoma (11.4% versus 3.9%; age- and sex-adjusted OR: 2.80; 95% CI: 1.26, 6.24). The association between peripheral arterial disease and higher IOP (age- and sex-adjusted mean, 16.4 vs 15.5; *P* = 0.05) was statistically marginal [6]. It is of importance that in Jeganathan´s study and our study, the associations with ABI were controlled for systemic factors such as hypertension, diabetes, body mass index, and serum triglyceride concentrations (multivariable-adjusted OR, 2.55; 95% CI, 1.09–5.98). Jeganathan et al. noted as a limitation of their study that the study population consisted of a large fraction of individuals with diabetes, what may have influenced the statistical analysis. Also, Shim and associates examined the role of systemic arterial stiffness in glaucoma patients with diabetes mellitus and used the brachial-ankle pulse wave velocity as a measure for arterial stiffness [12]. In a group of 75 diabetic glaucoma patients and 92 age-matched control individuals, a faster pulse wave velocity was related to a higher prevalence of glaucoma (OR: 3.74; 95% CI: 1.03, 13.56; *P* for trend = 0.036). One may take into account that in Jeganathan´s study the study sample was relatively small, that systemic parameters as potentially confounding factors were mostly not taken into account, and that the statistical significance of the association was relatively low.

In contrast to a study performed by Praveen and colleagues, we did not find any association between the ABI and the prevalence and degree of pseudoexfoliation in our study [9]. In Praveen´s hospital-based study on 160 patients aged 60+ years and with age-related cataract, the ABI was significantly lower in the pseudoexfoliative group as compared to the non-pseudoexfoliative group, and the presence of pseudoexfoliation increased the odds of a low ABI by a factor of 151. Limitations of Praveen´s study were the hospital-based recruitment of the study participants, the relatively small sample size, and that not all systemic parameters influencing the ABI might have been included into the multivariate analysis. In the study conducted by Jeon and associates, an association between a low ABI and retinal nerve fibre layer loss in patients with diabetes was found [16]. In that study on 167 individuals with diabetes type 2, a low Abi was associated with nerve fibre layer defects in the group aged 50+ years but not in the younger group [16]. Other studies assessed relationships between the brachial-ankle pulse wave velocity and the optic nerve head circulation [10], the prevalence of retinal vein occlusions [11], and retinal arteriolar narrowing [12].

The finding of our study that most of the major ocular diseases, such as age-related macular degeneration, glaucoma and cataract and some of their risk factors like intraocular pressure were not related with the ABI in the multivariable analysis suggests that these disorders and parameters are not markedly influenced by disorders associated with an abnormally low or abnormally high ABI. These observations may be of interest for the discussion of the aetiologies of the diseases in that arteriosclerosis may not be profoundly involved in the pathogenesis, if other systemic parameters such as age, sex, body mass index, waist-to-hip ratio, glucose serum concentration, prevalence of arterial hypertension and the mean systolic blood pressure have been taken into account.

Limitations of our study should be discussed. First, the quality of a population-based investigation markedly depends on the participation rate and the representativeness of the study population. With a participation rate of 80.5% of the eligible population, a major bias in the inclusion of the participants of our study may appear unlikely. Second, the group of individuals with available ABI measurements as compared with the group without ABI data was significantly younger, had a higher percentage of men versus women, and a longer axial length. The younger age and the preponderance of men might have led to an overestimation of the ABI, since younger age and male sex were associated with higher ABI measurements. It is however unlikely, that these differences had influenced the analysis of associations between the ABI and the prevalence and degree of ocular diseases. Third, arteriosclerosis may be associated more with retinal vessel-derived parameters or vascular disease such as retinal vein occlusion than with the other major ocular disease addressed in the present study. In a recent study, Wintergerst and colleagues reported on correlations between the ABI and the retinal and choriocapillaris vascular density as measured by optical coherence tomographic angiography [32]. Since however, we did not measure the diameters and tortuosity of the retinal vessels, we could not assess a correlation between arteriosclerosis, as measured by the ABI, and retinal vessel-derived variables. Fourth, abnormal ABI values are either low or high, so that taking the mean ABI for statistical analyses may cover significant relationships between ABI and other variables. It could mean that the ABI value might not have had a linear association with some variables. Plotting the ABI with the tested variables, such as thickness of the peripapillary retinal nerve fibre layer or the severity of diabetic retinopathy, however, did not show significant relationships between ABI and the tested parameters (Fig. 2). In addition, defining an abnormal ABI as an abnormally low ABI or an abnormally high ABI and performing a logistic binary regression analysis did not reveal significant associations between the prevalence of an abnormal ABI and the tested parameters.

Strengths of our investigation were that it was the first population-based investigation on the associations between ABI and ocular diseases with the inclusion of a relatively high number of systemic and ocular disorders into the multivariable analysis, so that a confounding effect due to unaddressed parameters may be relatively unlikely; that it was the first population-based investigation from Russia on ABI and its associations with systemic parameters; its relatively large study sample size, and that a multitude of systemic parameters was assessed and included into the statistical multivariable analysis.

In conclusion, in this ethnically mixed population from Russia, none of the major ocular diseases was associated with the ABI suggesting that subclinical atherosclerosis is not markedly associated with the aetiology of these ocular disorders.

### Summary

#### What was known before

- Only few studies with relatively few participants have assessed so far a potential association between the ankle-brachial blood pressure index and ocular diseases.

#### What this study adds

- None of the major ocular diseases was associated with ankle-brachial blood pressure index suggesting that subclinical atherosclerosis is not markedly associated with the etiology of these ocular disorders.

## Supplementary information

Supplementary Table 1
