# Supplementary material for: Ankle-brachial index and ocular diseases in a Russian population
Source: Eye (Lond). 2021 Nov 29;36(12):2294–303. doi: 10.1038/s41433-021-01846-x (PMC9674843; doi:10.1038/s41433-021-01846-x)
Supplement: Supplementary file 1 — Supplementary Table 1 [file 41433_2021_1846_MOESM1_ESM.docx]

Supplementary Table 1

Associations (multivariate analysis) between the ankle brachial index (ABI) (defined as the ratio of the highest values of ankle systolic blood pressure of both sides divided by the highest value of arm systolic blood pressure of both sides) and ocular parameters in the Ural Eye and Medical Study, after exclusion of the parameter of the mean systolic blood pressure (mean of measurements at both arms and both ankles)

| Parameter | Interval | Non-standardized Regression coefficient B | 95% Confidence Interval of B | *P*-Value | Variance Inflation Factor (VIF) |
| --- | --- | --- | --- | --- | --- |
| Sex | Men - Women | 0.03 | 0.02,0.05 | <0.001 | 1.28 |
| Body mass index | kg/m^2^ | -0.003 | -0.004, -0.001 | <0.001 | 1.25 |
| Waist-hip circumference ratio | Ratio | -0.09 | -0.16, -0.01 | 0.03 | 1.25 |
| Arterial hypertension, prevalence | Prevalence | -0.09 | -0.11, -0.07 | <0.001 | 1.09 |
| History of diarrhea | Prevalence | 0.14 | 0.02, 0.25 | 0.02 | 1.00 |
| History iron-deficiency anemia | Prevalence | 0.05 | 0.02, 0.08 | 0.02 | 1.05 |
| History of osteoarthritis | Prevalence | 0.02 | 0.003, 0.04 | 0.02 | 1.04 |
| Alcohol consumption, any | Prevalence | 0.04 | 0.02, 0.05 | <0.001 | 1.05 |
